# Robotic Sacro-Pelvic Fixation After Traumatic Fracture: A Case Report of the Utility of Robotics in Surgical Planning

**DOI:** 10.7759/cureus.92315

**Published:** 2025-09-14

**Authors:** Tyler M Cardinal, Eric Singh, Long Di, Timur Urakov

**Affiliations:** 1 Department of Neurological Surgery, University of Miami Miller School of Medicine, Miami, USA

**Keywords:** pelvic fixation, preoperative planning, robotic, spine surgery, trauma

## Abstract

The use of robotic technology in spine surgery has been shown to improve the accuracy of pedicle screw placement with faster operative time and less blood loss, particularly in the thoracolumbar region. Here we present a 22-year-old male who underwent successful robotic fixation of multiple unstable traumatic sacro-pelvic fractures. Given the complex nature of the construct, the robot facilitated advanced preoperative planning. Postoperatively, he did experience ileus likely due to opioid usage and his preoperative mechanical obstruction, which resolved quickly with medical management. He otherwise did very well after surgery and had resolution of his preoperative severe constipation. Our experience demonstrates the feasibility and utility of the robot in the fixation of sacropelvic fractures.

## Introduction

The introduction of robotic technology in spine surgery has revolutionized its safety and efficacy [1]. Numerous studies have shown that it improves the accuracy of pedicle screw placement compared to fluoroscopic placement [2-5]. Additionally, it allows for a minimally invasive surgical technique with faster operative time and less blood loss [3,5]. While robotic screw placement has proven its utility in the thoracolumbar spine, it may be particularly useful for surgical fixation of sacro-pelvic fractures, as it allows for preoperative visualization of screw placement via a minimally invasive approach. Here we present the case of a twenty-two-year-old male with multiple unstable traumatic sacro-pelvic fractures who underwent robotic lumbo-sacral and pelvic fixation.

## Case presentation

**Figure 1:**
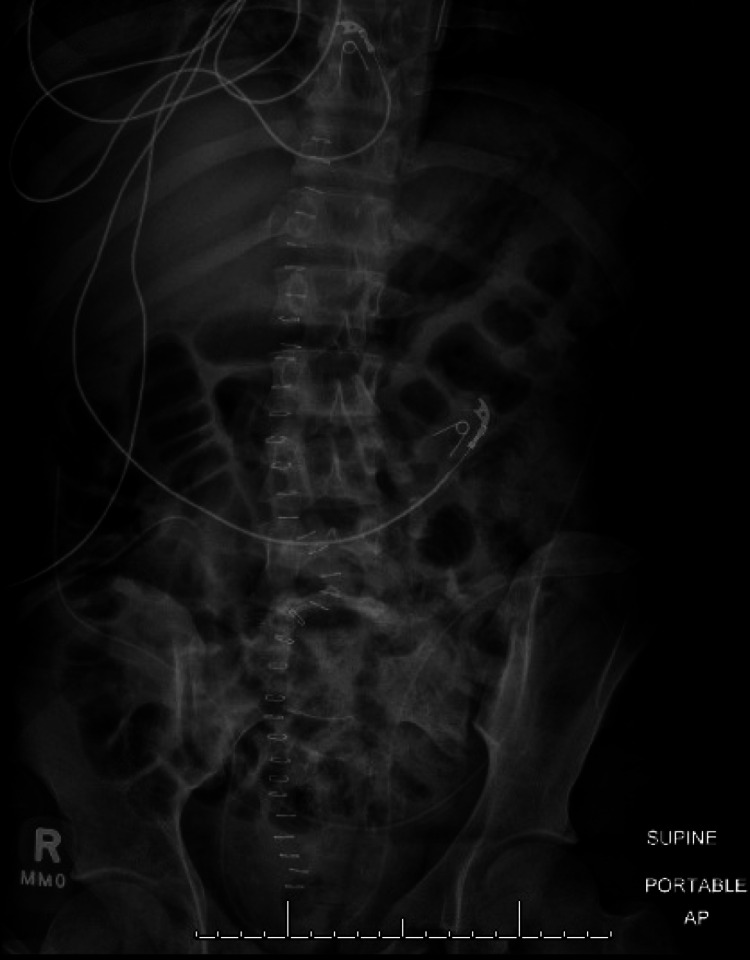
Pre-operative Sacral Fracture Anteroposterior X-ray from outside hospital demonstrating pre-sacral screw placement fracture

**Figure 2:**
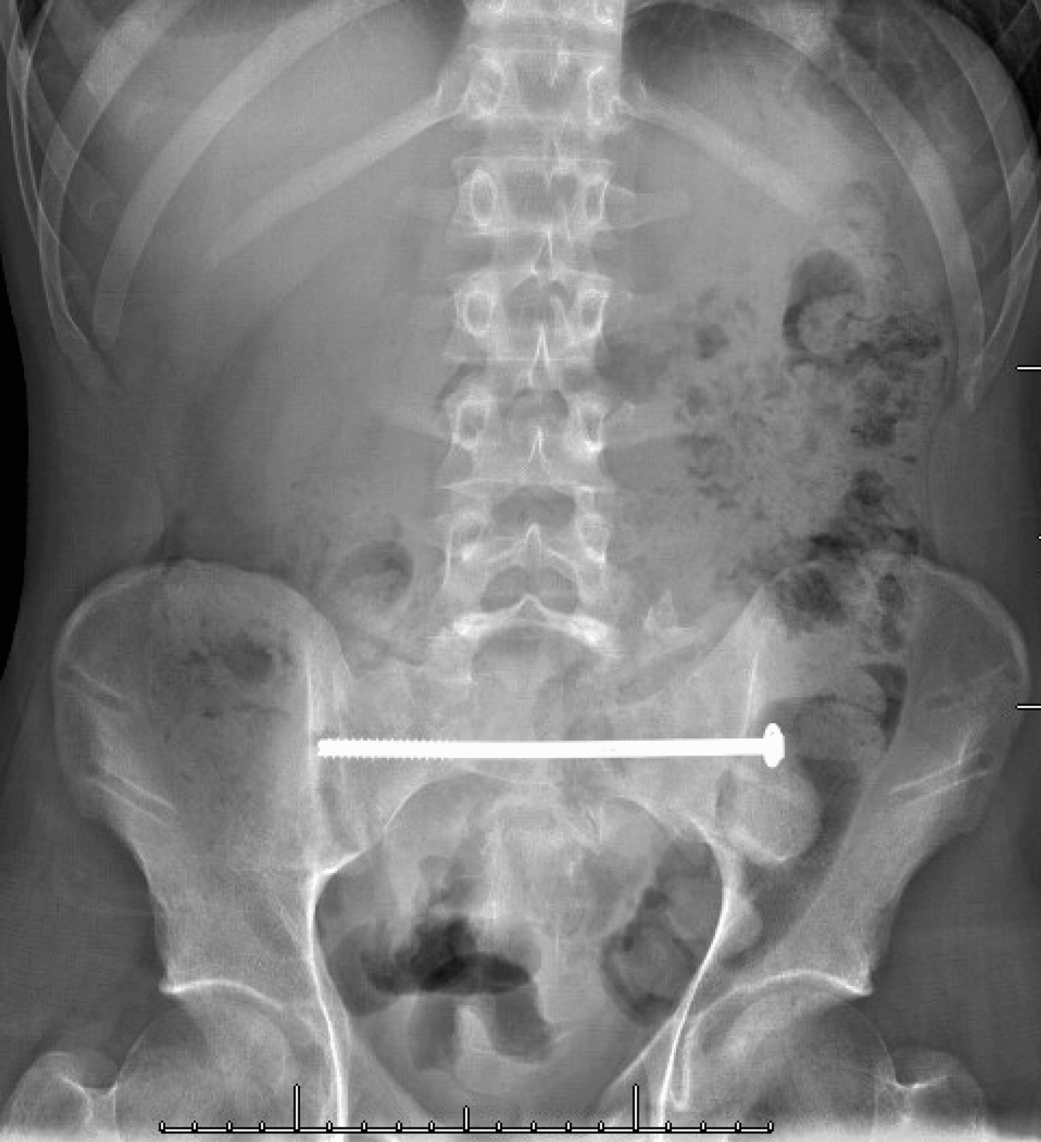
Initial Post-operative Sacral Screw Placement Anteroposterior X-ray from outside hospital demonstrating initial post-operative sacral screw placement

The patient is a 22-year-old male without any comorbidities who presented with traumatic, unstable pelvic and sacral fractures due to his car being hit by a train. He was transferred from an outside hospital after attempted surgical stabilization of his fractures with left-sided sacro-pelvic screw placement; however, he reported worsening tailbone pain that was uncontrolled by pain medications, as well as recurrent episodes of fecal impaction. On physical exam, he was full strength with decreased perianal sensation and normal rectal tone. Preoperative and postoperative imaging from outside the hospital was available, which showed malalignment of the left sacro-pelvic joint preoperatively (Figure 1) and subsequent reduction and fixation postoperatively (Figure 2).

MRI at that time demonstrated severe central stenosis throughout the sacral spine. Given concern for continued instability and significant nerve compression as the likely etiology for neurologic dysfunction and pain, the decision was made to return to the operating room. 

The patient was brought into the operating room, intubated, positioned prone on the Jackson table, prepped, and draped in the usual sterile manner. After the time-out, a low sacral midline incision was performed, and dissection was continued down to expose the L5 down to the S4 area as well as the bilateral iliac crests. We then placed the navigation array onto the spinous process of L4. The O‐arm was positioned appropriately around the patient, and lateral and AP fluoroscopy views confirmed that all necessary markers from the arrays would be contained within the spin. After O-Arm spin, L5-S4 instrumentation, as well as bilateral iliac bolts and S2 alar-iliac screws, were planned using the ExcelsiusGPS robot by Globus Medical (Audubon, PA) robotic system. Due to the fracture pattern, S2-4 screws could not be safely placed on the left side. The robot was draped in sterile fashion and brought into the field. Screws were placed utilizing robotic instrumentation. After screw placement, bony decompression was performed from S1-2. Rods and a system of side connectors were placed to secure all elements together. The bone was decorticated, and autograft and bone-morphogenic protein were layered over all exposed segments. Closure was performed with 0 Vicryl on the fascia, 2-0 Vicryl on the dermis, and 3-0 Monocryl on the skin.

The patient did well on postoperative day 0; however, he developed severe nausea/vomiting requiring naso-gastric tube placement and upgrade to the intensive care unit on postoperative day 1 for closer monitoring. This was likely due to an anesthetic effect temporarily inhibiting gastrointestinal motility and worsening his state of fecal impaction. This improved with serial enemas and an aggressive bowel regimen, and his diet was advanced. He was discharged on POD5, ambulating, tolerating regular diet, and had his first bowel movement in 10 days.

**Figure 3:**
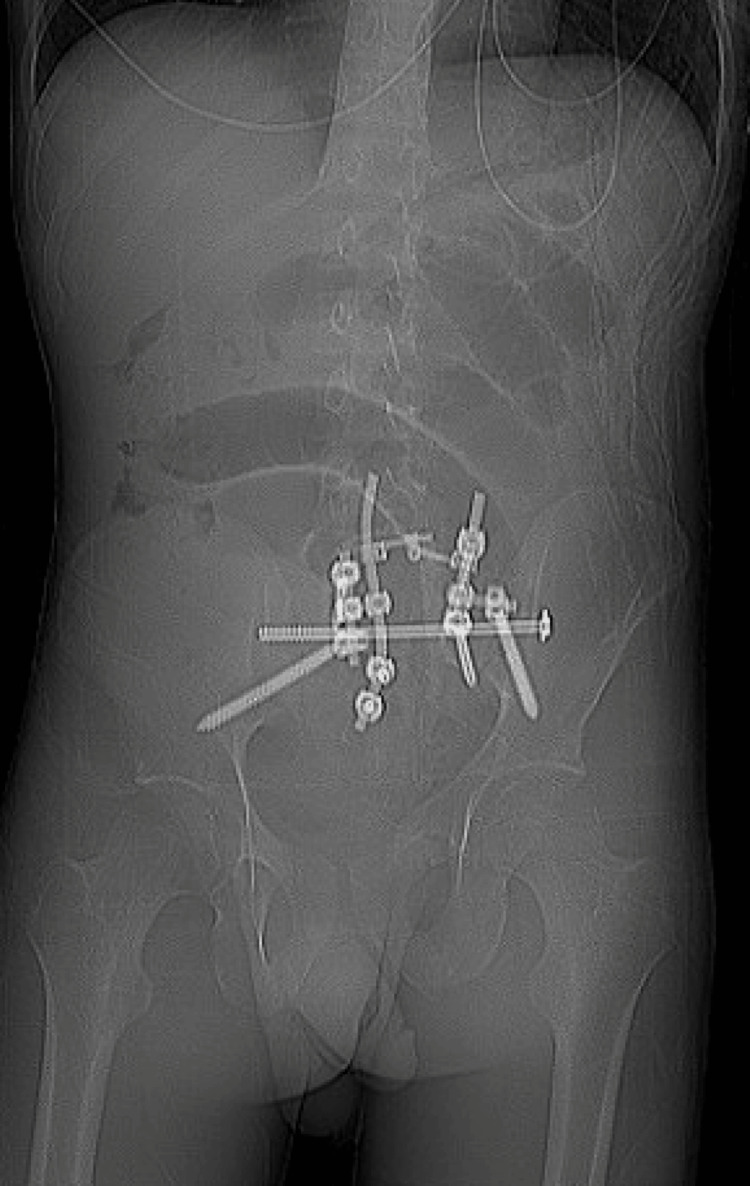
Follow-Up Post-operative X-ray Follow-up anteroposterior X-ray demonstrating hardware placement and bony fusion

At one-month postoperative visit, he continued to experience improvement in his preoperative severe back and leg pain and was ambulatory. At a four-month visit, he was having bowel movements without the use of laxatives and remained pain-free. His postoperative imaging demonstrated the beginnings of fusion of his fracture fragments with stable hardware placement (Figure 3).

## Discussion

Robotic-assisted instrumentation has emerged as a transformative approach in spine surgery, particularly for complex conditions such as sacral fractures. The case report of a young male who underwent robotic instrumentation for sacral fractures highlights the significant benefits of this technology. 

One of the primary advantages of robotic systems in spine surgery is their ability to enhance precision during instrumentation. The accuracy of robotic screw placement in spinal surgeries has been shown in multiple studies [2,3,6-8]. This is especially important in sacral fracture repair, with narrow corridors, and in this case, with prior instrumentation to fixate around. 

Robotic-assisted techniques often involve less invasive approaches compared to traditional methods, resulting in decreased soft tissue trauma and blood loss, with shorter hospitalization time [5,9]. In this case, we performed an open approach as we needed to decompress the sacral nerve roots; however, it is important to mention its utility as a minimally invasive technique in appropriate cases. 

Additionally, the use of robotic screw placement significantly limits the amount of radiation exposure for both the patient and the surgery team, compared to fluoroscopic instrumentation [5,8]. Liu et al. demonstrated in a 32-patient cohort that robotic surgery was associated with less blood loss, shorter surgical time, and shorter hospitalization with good outcomes when compared to a conventional open fixation [5]. 

There are numerous robotic spine systems out there, each with its own challenges. Robotic spine surgery is in its infancy, and more prospective studies are required to validate the workflow, usability, efficacy, and cost-effectiveness of the systems. Here, we discussed the case of a young patient with multiple sacral fractures and prior surgery at an outside hospital. We demonstrated the usefulness of robotic instrumentation in surgical planning and placement of hardware, particularly in complex cases like this one. We showed that this technique can be successfully performed with a good surgical outcome. 

## Conclusions

This case highlights the utility of robotic-assisted instrumentation in the surgical management of complex sacro-pelvic fractures. The ExcelsiusGPS robotic system facilitated preoperative planning and precise hardware placement despite the challenges of prior instrumentation and a disrupted anatomical corridor. While an open approach was necessary in this case due to the need for neural decompression, the use of robotic navigation enhanced surgical accuracy and safety. The patient demonstrated excellent postoperative recovery with resolution of neurological symptoms and stable hardware placement. Robotic-assisted spine surgery continues to evolve and shows promise for improving outcomes in complex spinal trauma, especially when careful planning and integration into traditional workflows are employed.
